# Conjunctival vascularization assessed by AS-OCTA as a biomarker of ocular involvement in primary Sjögren’s syndrome

**DOI:** 10.1093/rap/rkag074

**Published:** 2026-07-06

**Authors:** Benedetta Monosi, Sara Ferrigno, Isabella Corsi, Carlo Nucci, Maria Sole Chimenti, Francesco Aiello, Massimo Cesareo, Paola Conigliaro

**Affiliations:** Rheumatology, Allergology and Clinical Immunology, Department of Systemic Medicine, University of Rome Tor Vergata, Rome, Italy; Rheumatology, Allergology and Clinical Immunology, Department of Systemic Medicine, University of Rome Tor Vergata, Rome, Italy; Department of Experimental Medicine, Ophthalmology Unit, University of Rome Tor Vergata, Rome, Italy; Department of Experimental Medicine, Ophthalmology Unit, University of Rome Tor Vergata, Rome, Italy; Rheumatology, Allergology and Clinical Immunology, Department of Systemic Medicine, University of Rome Tor Vergata, Rome, Italy; Department of Experimental Medicine, Ophthalmology Unit, University of Rome Tor Vergata, Rome, Italy; Department of Experimental Medicine, Ophthalmology Unit, University of Rome Tor Vergata, Rome, Italy; Rheumatology, Allergology and Clinical Immunology, Department of Systemic Medicine, University of Rome Tor Vergata, Rome, Italy

**Keywords:** Sjögren’s syndrome, anterior segment-optical coherence tomography-angiography, dry eye

## Abstract

**Objectives:**

To determine whether conjunctival vascularization is increased in primary Sjögren’s syndrome (pSS) and whether it reflects systemic disease activity.

**Methods:**

Forty-one pSS patients and 39 matched healthy controls (HC) without ocular disease or dryness-inducing drugs underwent Schirmer test, tear break-up time (BUT), Oxford grading, Ocular Surface Disease Index (OSDI) and anterior segment OCT-angiography (AS-OCTA) of temporal conjunctiva. Conjunctival vessels were segmented on grey-scale images using a fixed threshold and expressed as vessel percentage. pSS also underwent a full rheumatologic assessment.

**Results:**

pSS patients had low systemic disease activity (median ESSDAI 0), long disease duration (median 9 years) and high symptom burden (ESSPRI 8). Conjunctival vascularization was significantly increased in pSS compared with HC (0.25 *vs* 0.13, *p* < 0.001). Tear function was reduced in pSS (lower Schirmer and BUT, higher OSDI and Oxford scores; all *p* < 0.001). An AS-OCTA cut-off ≥0.1941 optimally discriminated pSS from HC (AUC 0.88, sensitivity 94.9%, specificity 78.1%), outperforming Schirmer (AUC 0.76, sensitivity 88.9%, specificity 51.3%). About 78% of pSS patients had AS-OCTA ≥0.1941, compared with 10.2% of HC (*p* < 0.001). Subjects with pathological AS-OCTA values had lower Schirmer and BUT values, and higher OSDI scores (all *p* ≤ 0.001). Conjunctival vascularization weakly correlated with C3 levels (r=-0.36, *p* = 0.03), but not with C4 or other markers of systemic inflammation.

**Conclusion:**

AS-OCTA can effectively detect conjunctival vascular changes in pSS patients, offering an objective, non-invasive, and more sensitive method to assess eye dryness, reflecting the importance of a multidisciplinary management of the condition. Its potential link to systemic inflammation warrants further investigation.

Key messages

- AS-OCTA detects increased conjunctival vascularization in primary Sjögren’s syndrome patients, reflecting ocular dryness.
- Vascular changes on AS-OCTA effectively reflect tear film dysfunction and patient-reported dry eye symptoms.
- AS-OCTA outperforms traditional tear tests and may aid in the objective assessment of pSS-related dryness.

## Introduction

Primary Sjögren’s syndrome (pSS) is a chronic autoimmune disease resulting from a complex interplay between genetic predisposition and environmental factors that ultimately leads to immune dysregulation. The exocrine glands are the primary target of the disease, and the most widely accepted etiopathogenic theory is that of “autoimmune epithelitis” [1], in which infiltrating lymphocytes cause chronic glandular inflammation [2, 3]. In the conjunctiva, this pathogenetic mechanism leads to loss of goblet cells and squamous metaplasia, whereas in the cornea leads to focal epithelial loss and exposure of Bowman’s membrane. Both the quantity and quality of the tear film are affected, as the aqueous component is reduced, and the lipid layer is compromised when meibomian glands dysfunction is present. These alterations result in decreased tear film stability and accelerated evaporation [4]. Symptoms include burning, grittiness, itching, redness, photophobia, and an inability to cry, while the most significant complications are conjunctival bacterial infections, corneal abrasions, and ulcers. Primary SS is also characterized by extra-glandular manifestations, and patients can be classified into three subgroups: cluster 1, characterized by B cell activation and low symptom burden (BALS); cluster 2, with high systemic disease activity (HSA); and cluster 3, with low systemic disease activity but high symptom burden (LSAHS) [5]. Interestingly, it has been found that patients with vision-threatening ocular findings are 3.9 times more likely to have systemic involvement [6].

Ocular dryness is assessed using several diagnostic tests: the Schirmer test evaluates tear production, the tear film break-up time (BUT) test assesses tear film quality, and the ocular staining score (OSS) quantifies epithelial damage of the cornea and conjunctiva using lissamine green or fluorescein staining, respectively [7].

Optical coherence tomography angiography (OCTA) is a non-invasive imaging technique that uses laser light reflectance from moving red blood cells to depict vessels within different segmented ocular areas. By imaging the same tissue area repeatedly, OCTA identifies areas of high, low, or absent blood flow [8]. It has been primarily used to visualize the microvasculature of the retina and choroid in systemic diseases including diabetes, hypertension, and chronic kidney disease. In Systemic Lupus Erythematosus (SLE), OCTA has demonstrated reduced retinal vessel density compared with healthy controls (HC) [9], correlating with renal function and histological vascular damage in lupus nephritis [10]. Accordingly, in pSS OCTA has demonstrated reduced retinal vessel density in the posterior segment of the eye [11].

However, more recently, OCTA has been applied to the anterior segment (AS-OCTA) to detect pathological corneal neovascularization in diabetic retinopathy and hypervascularization in inflammatory and autoimmune conditions such as anterior uveitis, scleritis, and pSS [12, 13]. Thus, the aim of the present study was to determine whether conjunctival vascularization is increased in pSS patients reflecting ocular dryness, and whether this increase correlates with systemic disease activity.

## Methods

### Study design and population

This was a monocentric, observational, cross-sectional study conducted at the Rheumatology and Ophthalmology Units of Policlinico Tor Vergata, Rome, Italy, between 1st November 2022 and 30th September 2023. The study enrolled 41 consecutive patients with a confirmed diagnosis of pSS, according to the 2016 ACR/EULAR classification criteria [14]. A control group of 39 age- and sex-matched HC with no history of autoimmune or ocular disease was also recruited. Demographic data were collected for all participants.

Inclusion criteria were: 1) age between 18 and 80 years; 2) best-corrected visual acuity (BCVA) greater than 0.5 LogMAR. Exclusion criteria were: 1) coexistence of other rheumatic diseases, such as Antiphospholipid Syndrome (APS), SLE, Systemic Sclerosis, Mixed Connective Tissue Disease (MCTD), or Rheumatoid Arthritis (RA); 2) retinal toxicity due to antimalarials drugs, as defined by the 2016 American Academy of Ophthalmology criteria [15]; 3) primary ophthalmic pathology, including scleritis, episcleritis, lens opacities, drusen-like deposits, focal atrophy, retinal pigment epithelium detachment, or a history of ocular trauma or surgery; 4) use of topical ophthalmologic medications (except for artificial tears); 5) use of systemic medications associated with ocular dryness; and 6) use of contact lenses.

All participants provided written informed consent prior to enrolment. The study protocol was approved by the Scientific Ethics Committee of Policlinico Tor Vergata, Rome, Italy (protocol no. 263/22), and was conducted in accordance with the ethical standards of the Declaration of Helsinki (last updated in 2008).

### Rheumatological evaluation

All patients with pSS underwent a comprehensive rheumatologic evaluation. Laboratory parameters were assessed as follows: anti-Ro/SSA 60 and 52 kDa and anti-La/SSB (U/ml) were measured using a commercial enzyme-linked immunosorbent assay (ELISA QUANTA LiteTM; Inova Diagnostics); antinuclear antibodies (ANA ≥ 1:160) were evaluated by direct immunofluorescence on Hep-2 cells (QUANTA LiteTM; Inova Diagnostics); Rheumatoid Factor (RF) levels were determined by nephelometry; cryoglobulin levels were assessed by cryoprecipitation; and plasma complement components C3 and C4 (mg/dl; normal values: 83–193 and 15–57 respectively) were measured using immunoturbidimetry. Additional laboratory tests included erythrocyte sedimentation rate (ESR, mm/h), C-reactive protein (CRP, mg/l), immunoglobulin levels (IgG, IgA, IgM; mg/dl), and beta-2 microglobulin (mg/dl).

Systemic disease activity was evaluated using the EULAR Sjögren’s Syndrome Disease Activity Index (ESSDAI), whereas patient-reported symptom burden was assessed with the EULAR SS Patient-Reported Index (ESSPRI), which measures dryness, fatigue, and pain [16]. Disease duration and ongoing treatments with prednisone (PDN) ≥5 mg daily, hydroxychloroquine (HCQ), or conventional immunosuppressants (azathioprine, methotrexate, ciclosporin, mycophenolate, cyclophosphamide) were also recorded.

Finally, patients were categorized into three clusters, as previously described [5]: Cluster 1, characterized by low systemic disease activity and low symptom burden (ESSDAI <5 and ESSPRI <5); Cluster 2, comprising patients with high disease activity (ESSDAI ≥5); and Cluster 3, including patients with low systemic disease activity but high symptom burden (ESSDAI <5 and ESSPRI ≥5).

### Ophthalmological evaluation

Patients and HC underwent a comprehensive ophthalmologic assessment, performed by an experienced ophthalmologist. The evaluations included: 1) Schirmer type-1 test, performed without topical anaesthesia, using a 30–35 mm × 5 mm bibulous paper strip placed in the lower conjunctival fornix of the temporal side of both eyes for 5 min. Values ≤5 mm were considered indicative of pathological tear production. 2) Slit-lamp BUT test, instilling fluorescein dye into the lower fornix. Patients were asked to blink several times and then keep their eyes open. The interval between the last blink and the appearance of the first dry spot on the cornea was measured. A BUT <10 s was considered pathological. The definition of short BUT (sBUT) was also applied for values ≤5 s. 3) Ocular surface fluorescein staining, performed and graded according to the Oxford Scheme [17]. 4) Ocular Surface Disease Index (OSDI) questionnaire, administered to patients and controls to assess subjective eye discomfort [18].

#### Anterior segment optical coherence tomography angiography

AS-OCTA was performed in both pSS patients and HC using the Avanti AngioVue Imaging System (Optovue, Inc., software version 2018.1.0.22, Fremont, CA). Evaluation of conjunctival vascularization was achieved by manually adjusting the focus with a defocus of +20 diopters (scan protocol: manual adjustment, defocus +20 diopters). An external fixation light was positioned on the nasal side of the examined eye to optimize exposure of the temporal conjunctiva during scanning. Normal blinking was allowed during the procedure.

A 3x3mm scan of the temporal conjunctiva was acquired by an experienced operator. The conjunctival vascular depth settings were manually adjusted, with a reference depth of 1,200 μm applied to exclude scleral vessels. Images were excluded from analysis if their signal strength index was <50, if they showed significant motion artifacts due to poor fixation, or if eyelash shadows caused occlusion [19]. The resulting enface images were processed using MATLAB software and converted to grayscale, with pixel intensity values ranging from 0 to 255. Analysis of images with threshold values above 110 pixels showed a loss of definition of the thinner vessel component; conversely, the images analyzed with threshold values below 110 pixels resulted in an artificial increase in the vessel component due to an increase in background noise. Therefore, a cut-off of 110 pixels was established to define the vascular component, and the percentage of vascular density was calculated to measure the amount. This approach is consistent with standard image binarization methods used in vascular segmentation [19].

### Statistical analysis

Continuous variables were expressed as mean ± standard deviation (SD) or as median and interquartile range (IQR), after assessing their distribution with the Shapiro-Wilk test or the D’Agostino and Pearson omnibus test. Categorical variables were expressed as counts and percentages. Categorical variables were compared using the χ2 test. Continuous variables were compared using the parametric unpaired t-test or the nonparametric Mann–Whitney U test, as appropriate. The significance of correlations was assessed using Pearson’s correlation coefficient or Spearman’s rank correlation coefficient, depending on data distribution. A *P*value <0.05 was considered significant. Receiver Operating Characteristic (ROC) curve analysis was performed in pSS and HC groups to evaluate the diagnostic accuracy of AS-OCTA, Schirmer and BUT tests. For AS-OCTA, cut-off values were selected based on the best likelihood ratio (LR) and the highest sensitivity and specificity. For the Schirmer and BUT tests, cut-off values currently used to detect ocular dryness in pSS were applied. Statistical analyses were performed using R (version 4.4.1) and GraphPad Prism (version 8 for iOS).

## Results

A total of 41 pSS patients and 39 HC were enrolled in the study. Demographic and clinical characteristics of the study population are reported in Table 1. There were no significant differences in sex or mean age between pSS patients and HC. Patients with pSS had low disease activity [ESSDAI 0 (0–3)], with a median disease duration of 9 (4–16) years. The median ESSPRI score was 8 (6–9). Regarding ongoing treatments, 25 patients (61%) were receiving HCQ, while 10 patients (24.4%) were on immunosuppressive therapy (azathioprine, methotrexate, leflunomide). Only 7 patients (17%) were being treated with PDN.

**Table 1.** Characteristics of primary Sjögren’s Syndrome patients (pSS) and healthy controls (HC).

		pSS n = 41	HC n = 39	p-value
Female sex		40 (98)	34 (87)	0.181
Age (years)		58 (±13)	59 (±6.6)	0.804
Disease duration (years)		9 (4-16)	NA	–
Autoantibodies	ANA	36 (88)	NA	–
	anti-SSA/Ro (U/ml)	26 (63)	NA	–
	anti-SSB/La (U/ml)	12 (29)	NA	–
	RF (U/ml)	9 (22)	NA	–
Gammaglobulins (%)		17 ± 4.2	NA	–
CRP (mg/l)		0.09 (0-0.4)	NA	–
ESR (mm/h)		22 (11-30)	NA	–
IgA (mg/dl)		227 ± 114	NA	–
IgG (mg/dl)		1213 ± 421	NA	–
IgM (mg/dl)		122 (63-165)	NA	–
C3 (mg/dl)		114 ± 23	NA	–
C4 (mg/dl)		23 ± 8	NA	–
Beta-2 Microglobulin (mg/l)		0.26 (0.18-1.46)	NA	–
ESSDAI		0 (0-3)	NA	–
ESSPRI		8 (6-9)	NA	–
Clusters	Cluster 1	3 (7.9%)	NA	–
	Cluster 2	6 (15.8)	NA	–
	Cluster 3	29 (76.3)	NA	–
Treatments	PDN	7 (17)	NA	–
	HCQ	25 (61)	NA	–
	IS	10 (24.4)	NA	–
Ocular evaluation	Schirmer test	5 (2.8-9.3)	10 (8.4-12)	**<0.001**
	BUT	5 (3-7.8)	10 (10-10)	**<0.001**
	OSDI	56 (38-73)	11 (10-20)	**<0.001**
	Oxford >1	22 (53.6)	2 (5.1)	**<0.001**
	AS-OCTA	0.25 ± 0.09	0.13 ± 0.05	**<0.001**

ANA: Antinuclear antibody; RF: Rheumatoid Factor; CRP: C-Reactive Protein; ESR: Erythrocyte Sedimentation Rate; ESSDAI: EULAR Sjögren’s Syndrome disease activity index; ESSPRI: EULAR Sjögren’s Syndrome Patient-Reported Index; PDN: Prednisone; HCQ: Hydroxychloroquine; IS: immunosuppressant; BUT: Break Up Time; OSDI: Ocular Surface Disease Index; AS-OCTA: Anterior Segment-Optical coherence tomography-angiography. Continuous variables are presented as mean ± standard deviation or as median and interquartile range and are compared using the parametric unpaired t-test or the nonparametric Mann–Whitney U test, as appropriate. Categorical variables are presented with absolute frequencies and percentages and are compared using the Chi-squared test or Fisher’s exact test, as appropriate. A *P*value <0.05 is considered statistically significant.

**Figure 1:**
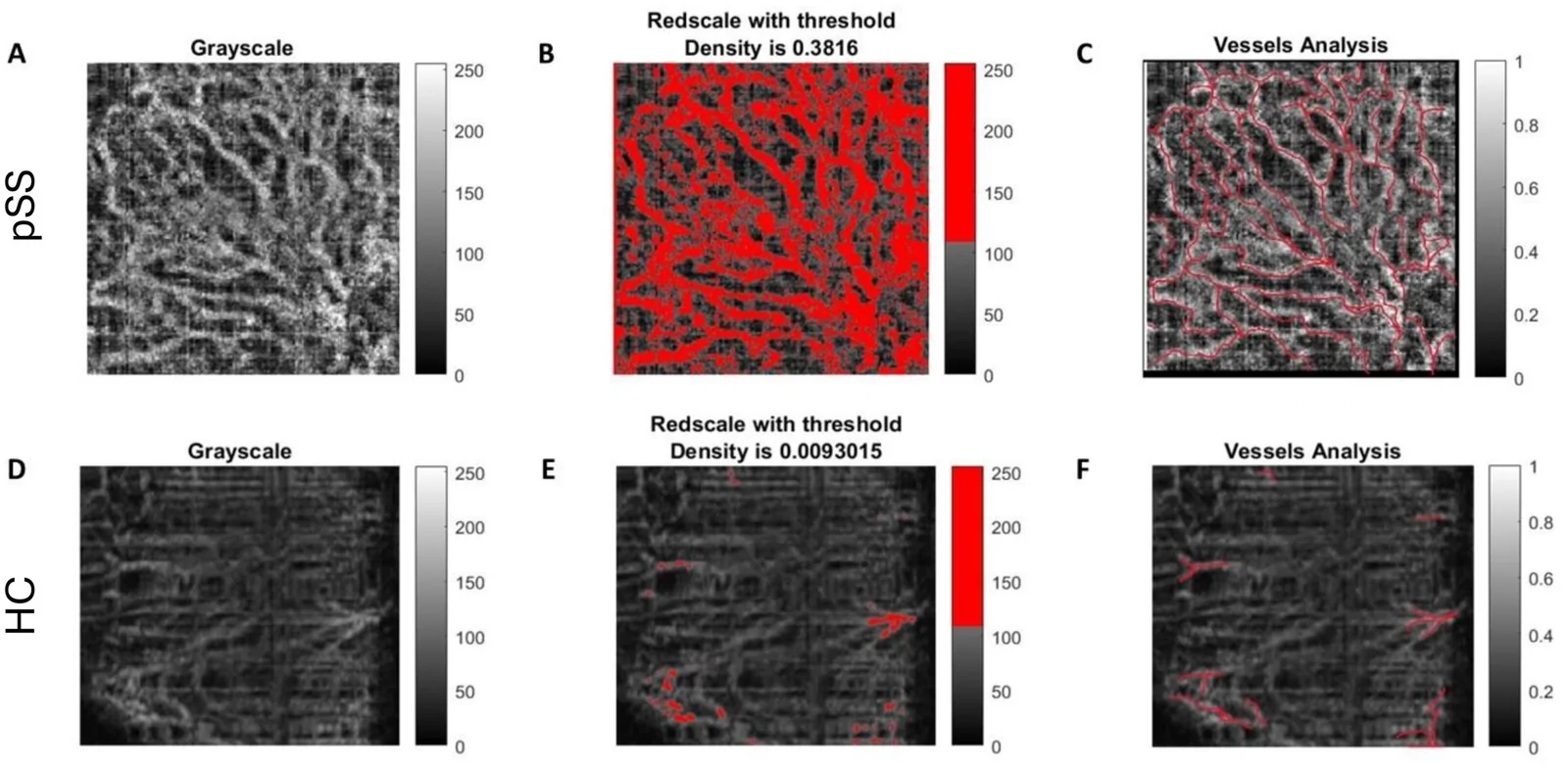
Anterior Segment OCTA images in primary Sjögren’s Syndrome patients (pSS) and healthy controls (HC). Primary SS patients (panels A-C) display increased conjunctival vascularization than HC (panels D-F), shown through grayscale, redscale, and combined density maps.

Regarding ophthalmological data, mean AS-OCTA values were significantly higher in pSS patients compared with HC (0.25 ± 0.09 *vs* 0.13 ± 0.05, *P* < 0.001) (Table 1 and Fig. 1). As expected, Schirmer and BUT test values were significantly lower in pSS than in HC [5 (2.8–9.3) *vs* 10 (8.4–12), *P* < 0.001 and 5 (3–7.8) *vs* 10 (10–10), *P* < 0.001, respectively], while OSDI scores were higher in pSS compared with HC [56 (38–73) *vs* 11 (10–20), *P* < 0.001]. Additionally, a significantly greater proportion of pSS patients had an Oxford score >1 compared with HC (53.6% *vs* 5.1%, *P* < 0.001) (Table 1). Mean values of AS-OCTA, Schirmer test, and BUT from both eyes were used for the analysis, as no significant inter-eye differences were observed (data not shown).

**Figure 2:**
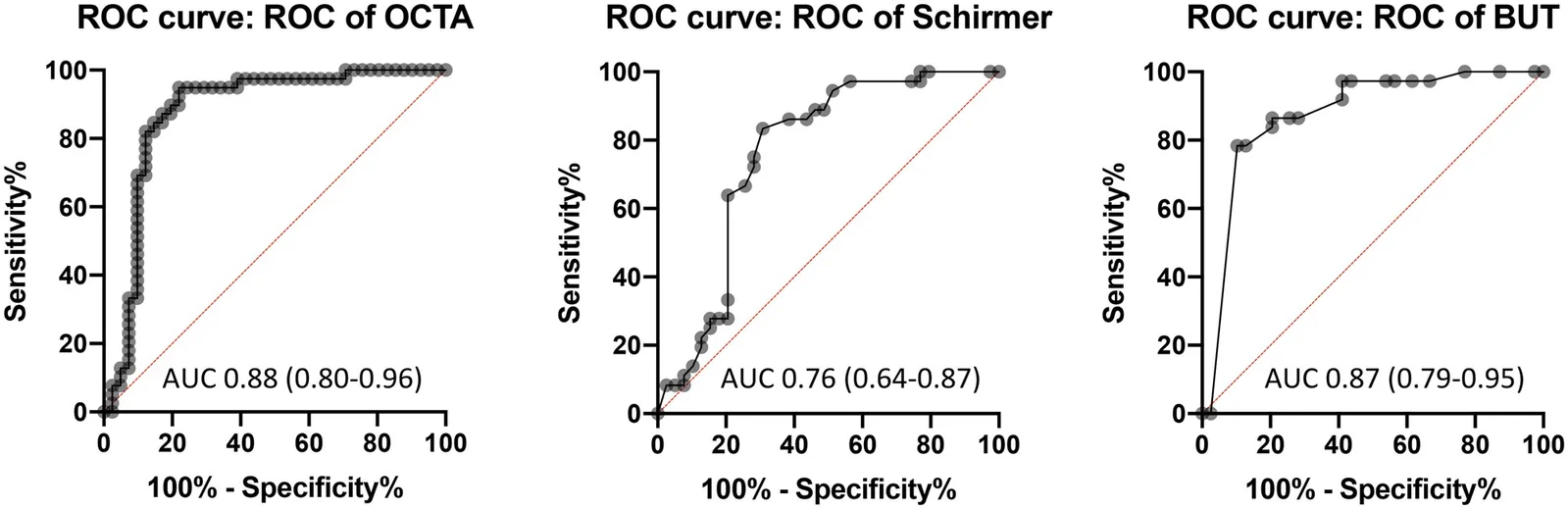
Receiver operating characteristic (ROC) curves and corresponding areas under the curve (AUC) for AS-OCTA, Schirmer test, and tear film break-up time (BUT)

The ROC curve and corresponding area under the curve (AUC) demonstrated high diagnostic accuracy of AS-OCTA using a cut-off of ≥0.1941, with an AUC of 0.88 (95% CI: 0.80–0.96), sensitivity of 94.87%, specificity of 78.05%, and a LR of 4.32. The same analysis performed for Schirmer and BUT tests, using a cut-off of <5.25 for the former and ≤9.7 for the latter, showed an AUC of 0.76 (95% CI: 0.64–0.87), sensitivity of 88.9%, specificity of 51.3%, LR of 1.82 for the Schirmer test, and an AUC of 0.87 (95% CI: 0.79–0.95), sensitivity of 78.4%, specificity of 89.7%, and LR of 7.6 for the BUT test (Fig. 2).

**Figure 3:**
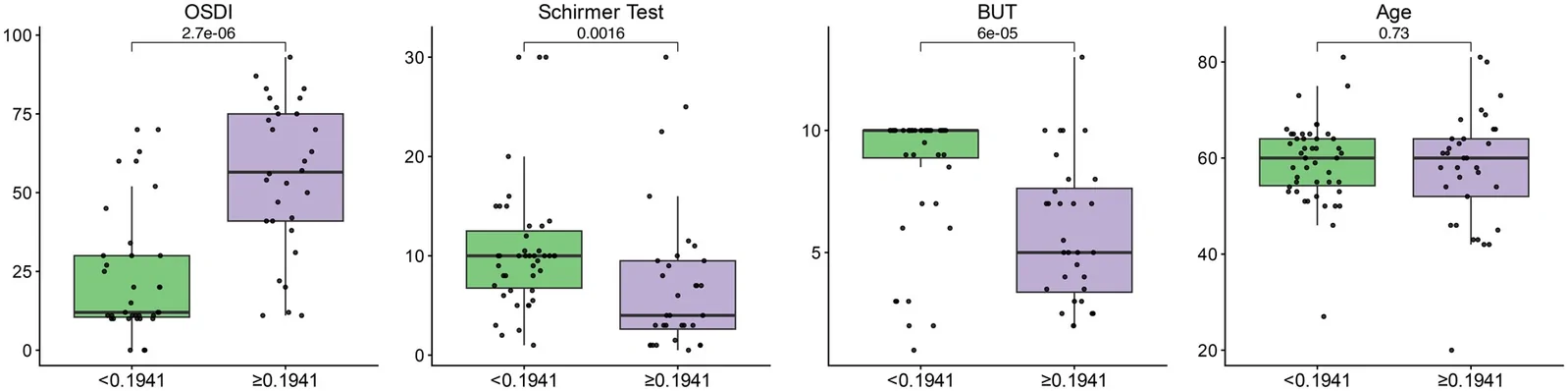
Boxplots comparing OSDI, Schirmer test, tear film break-up time (BUT), and age between subjects with normal (<0.1941) and pathological (≥0.1941) AS-OCTA vascularization

AS-OCTA data from pSS patients and HC were categorized according to the selected cut-off. A total of 32 (78%) pSS patients had AS-OCTA ≥0.1941, compared with only 4 (10.2%) HC (*P* < 0.001). To further support the pathological significance of increased AS-OCTA vascularization, we compared other ophthalmologic tests in the entire study population (pSS and HC combined), stratified by AS-OCTA cut-off. As shown in Fig. 3, subjects with pathological AS-OCTA values had significantly lower Schirmer and BUT test values (*P* = 0.0016 and *P* < 0.001, respectively), and significantly higher OSDI scores (*P* < 0.001). Age and sex had no impact on AS-OCTA vascularity (*P* = 0.73 and *P* = 1, respectively).

**Figure 4:**
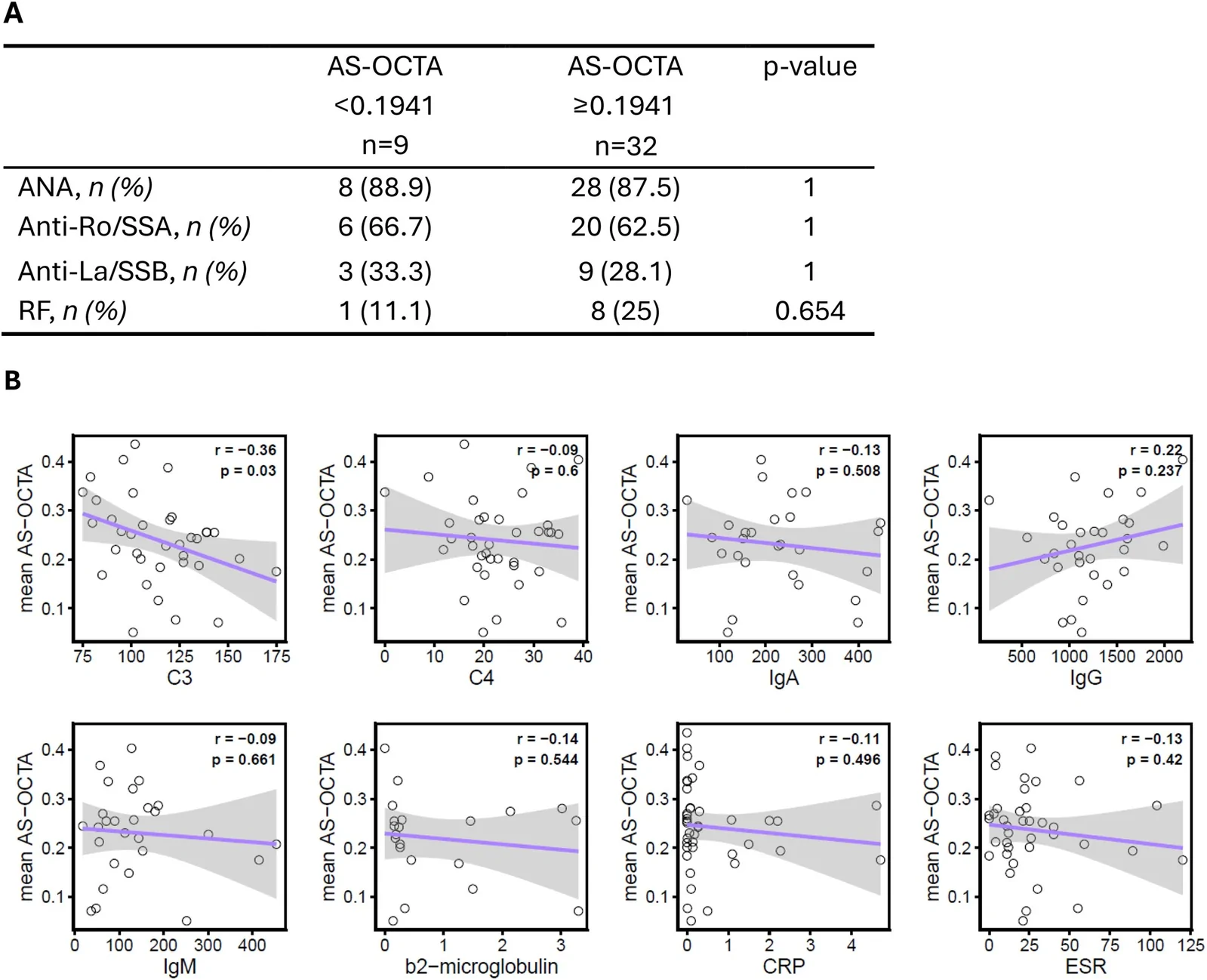
AS-OCTA values and disease activity markers in patients with primary Sjögren’s syndrome. **(A)** Autoantibody positivity according to AS-OCTA cut-off of 0.1941; **(B)** Correlation between mean AS-OCTA values and laboratory parameters of systemic disease activity

Regarding pSS-specific autoantibodies, the positivity for ANA, anti-Ro/SSA, anti-La/SSB, or RF was not significantly different between patients with AS-OCTA values <0.1941 and ≥0.1941 (Figure 4A). Moreover, biomarkers of disease activity, including levels of IgA, IgG, IgM, b2-microglobulin, ESR, and CRP, did not show any significant association with mean AS-OCTA values (Figure 4B). AS-OCTA values correlated significantly with C3 levels (r=-0.36, *P* = 0.03), but not with C4 levels (r=-0.09, *P* = 0.6) (Figure 4B).

**Figure 5:**
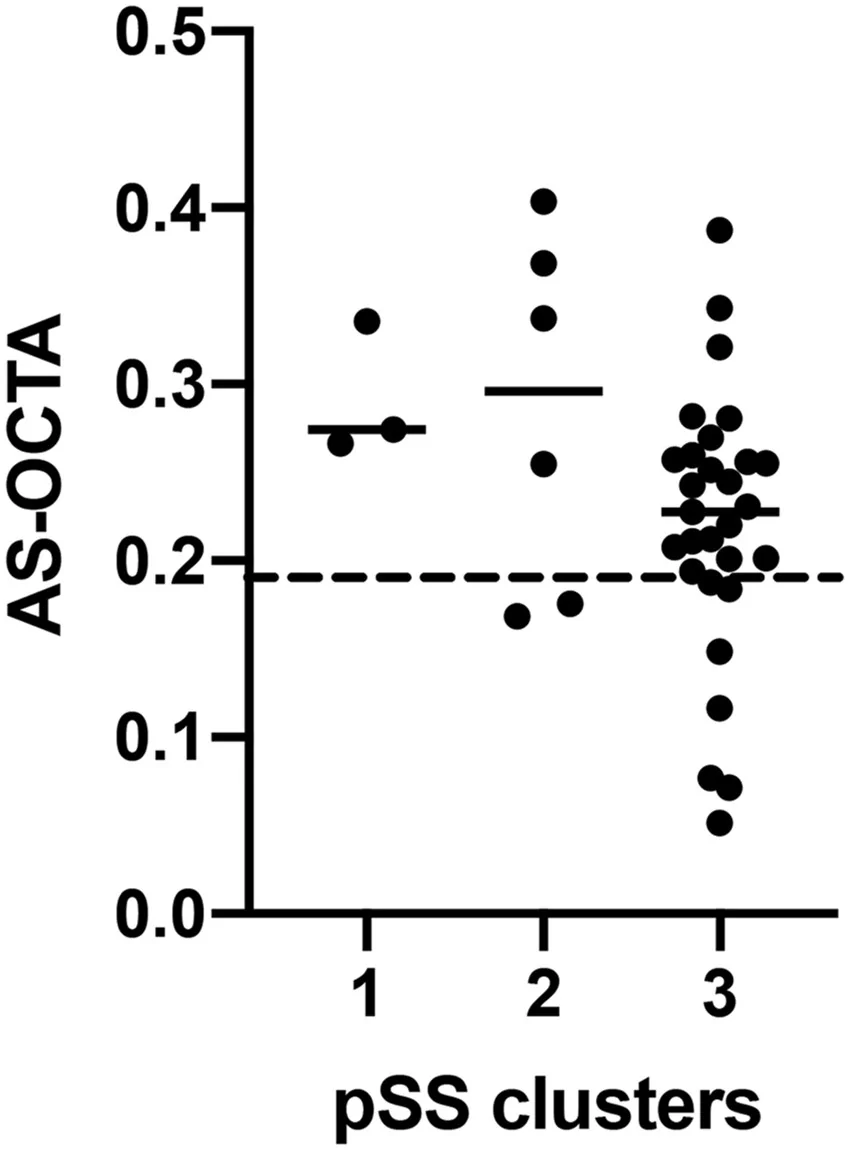
Distribution of AS-OCTA values across clinical clusters of patients with primary Sjögren’s syndrome

Finally, patients were stratified according to the three previously described clusters [5]. Although no statistically significant differences in AS-OCTA values were observed between the subgroups, cluster 2 showed higher vascularization compared with clusters 1 and 3, as illustrated in Fig. 5.

## Discussion

Our findings demonstrate increased conjunctival vascularization in patients with primary Sjögren’s syndrome (pSS) compared with age- and sex-matched healthy controls, reflecting impaired tear film production and the heightened ocular discomfort characteristic of this condition. While anterior segment optical coherence tomography angiography (AS-OCTA) effectively detected these vascular changes, the relationship between anterior segment microvascular alterations and systemic disease activity remains to be fully clarified.

More than 95% of patients with pSS experience sicca symptoms [20], and dry eye represents a major determinant of reduced quality of life, often exceeding the impact of systemic manifestations [21]. Chronic tear deficiency promotes ocular surface damage, including squamous metaplasia, underscoring the importance of early recognition and treatment. Current EULAR recommendations advocate a primarily symptomatic approach, with artificial tears as first-line therapy, and recommend caution regarding the short-term use of topical corticosteroids and non-steroidal anti-inflammatory drugs due to their potential side effects [22]. Although topical ciclosporin can improve tear production and ocular surface stability [23], no treatment can reverse glandular dysfunction. Consistently, the 2016 ACR/EULAR classification criteria incorporate objective measures of dryness, such as Schirmer test and ocular staining score, emphasizing the central role of glandular involvement in disease classification [15]. Our cohort showed significantly reduced tear film production, shorter tear film break-up time (BUT), and greater ocular discomfort compared with controls.

Ocular surface inflammation represents a key mechanism underlying dry eye in pSS. Pro-inflammatory cytokines, such as interferon-gamma (IFN-γ), interleukin(IL)-17, IL-1β, IL-2, IL-4, IL-6, and IL-8, accumulate in the conjunctiva [24] and tear film [25]. In the conjunctiva, IFN-γ, TNF-α and IL-1β regulate the secretion of angiogenic vascular endothelial factor (VEGF), promoting vessel growth [24]. A similar VEGF pathway, in particular the activation of VEGF-A and its receptor 2 (VEGFR2) pathway, has been described in salivary glands, supporting the concept that aberrant neovascularization is part of the broader inflammatory process in pSS [26]. Although OCTA has been widely applied to the posterior segment in systemic [27–29] and autoimmune diseases [9–11], recent evidence suggests its use in the anterior segment [12, 30, 31]. Our results confirm the reported increased conjunctival microvascular density in pSS [13] and support AS-OCTA as a non-invasive tool for quantifying these changes. Notably, we propose a potential diagnostic cut-off value for pathological anterior segment vascularization.

In terms of diagnostic performance, AS-OCTA demonstrated high sensitivity (94.87%), exceeding that of the Schirmer test (88.9%) and BUT (78.4%), suggesting strong ability to detect true positive cases. However, sensitivity and specificity must always be interpreted together to obtain a comprehensive view of diagnostic performance. When considering specificity, AS-OCTA (78.05%) outperformed the Schirmer test (51.3%) but was slightly lower than BUT (89.7%). This indicates that AS-OCTA is particularly useful for ruling out dryness when the test result is negative, while still offering reasonable power to confirm the condition when positive. Importantly, unlike traditional tear function tests, AS-OCTA measurements were not influenced by age, which may represent a significant advantage given the physiological decline in tear secretion and stability over time [32–34]. Overall, the combination of high sensitivity and moderate specificity supports AS-OCTA as a reliable and comprehensive diagnostic tool for dry eye assessment in pSS. However, cost, availability, and the need for validation in independent cohorts may limit its immediate widespread implementation.

Chronic inflammation may contribute not only to conjunctival vascular changes, but also to microvasculature damage in other organs [35, 36]. Microvessel density has been reported to increase with the severity of inflammatory lesions in minor salivary glands of pSS patients [26], suggesting that angiogenesis plays an important role in disease activity and progression [37]. In the context of systemic disease activity, AS-OCTA values showed a weak inverse correlation with C3 levels, while no significant associations were observed with other clinical or laboratory markers. Hypocomplementemia in pSS has been associated with more severe serological profiles, increased systemic involvement, and poorer outcomes [38–40], raising the possibility that conjunctival vascular alterations may partially reflect systemic inflammatory burden. Although most data derive from minor salivary gland biopsy studies, in which the severity of glandular involvement appears to correlate with systemic manifestations [41–43], recent evidence suggests that ocular involvement may similarly reflect systemic inflammatory burden [44]. A recent study correlated higher concentrations of inflammatory cytokines in tear fluid of pSS with both severe ocular surface alterations and higher ESSDAI scores [45], further supporting a link between local ocular changes and systemic disease activity. Nevertheless, the correlations observed in our study were modest, and their clinical significance remains uncertain.

Subgroup stratification revealed a trend toward higher AS-OCTA values in patients with higher disease activity, although the limited sample size within clusters reduces the robustness of this finding. Most patients belonged to the low-activity, high-symptom subgroup, restricting our ability to evaluate vascular changes in more severe systemic phenotypes. Moreover, the absence of previous literature examining the relationship between systemic disease activity and AS-OCTA further limits our conclusions. Therefore, our findings should be considered exploratory and larger, longitudinal studies are needed to confirm this association and clarify its pathophysiological significance. Furthermore, the cross-sectional design precludes assessment of longitudinal changes or treatment responsiveness. The potential influence of immunosuppressive therapy on anterior segment vascularization also warrants further investigation.

In conclusion, AS-OCTA enables detection of conjunctival vascular alterations in pSS, reflecting glandular tear film impairment. This imaging modality may enhance diagnostic precision and contribute to earlier identification of ocular involvement to prevent potentially dangerous ocular complications, supporting a multidisciplinary approach to disease management. Larger, longitudinal studies are needed to confirm its prognostic value and clarify its relationship with systemic disease activity.

## Data Availability

The data that support the findings of this study are available from the corresponding author upon reasonable request.

## Notes

*Disclosure statement:* The authors have declared no conflicts of interest.
